# An Expedient Synthesis, Acetylcholinesterase Inhibitory Activity, and Molecular Modeling Study of Highly Functionalized Hexahydro-1,6-naphthyridines

**DOI:** 10.1155/2015/965987

**Published:** 2015-01-29

**Authors:** Abdulrahman I. Almansour, Raju Suresh Kumar, Natarajan Arumugam, Alireza Basiri, Yalda Kia, Mohamed Ashraf Ali

**Affiliations:** ^1^Department of Chemistry, College of Science, King Saud University, P.O. Box 2455, Riyadh 11451, Saudi Arabia; ^2^School of Pharmaceutical Sciences, Universiti Sains Malaysia, 11800 Minden, Penang, Malaysia; ^3^School of Chemical Sciences, Universiti Sains Malaysia, 11800 Minden, Penang, Malaysia; ^4^Pharmacogenetic and Novel Therapeutic Institute for Research in Molecular Medicine, Universiti Sains Malaysia, 11800 Penang, Malaysia

## Abstract

A series of hexahydro-1,6-naphthyridines were synthesized in good yields by the reaction of 3,5-bis[(*E*)-arylmethylidene]tetrahydro-4(1*H*)-pyridinones with cyanoacetamide in the presence of sodium ethoxide under simple mixing at ambient temperature for 6–10 minutes and were assayed for their acetylcholinesterase (AChE) inhibitory activity using colorimetric Ellman's method. Compound **4e** with methoxy substituent at *ortho*-position of the phenyl rings displayed the maximum inhibitory activity with IC_50_ value of 2.12 *μ*M. Molecular modeling simulation of **4e** was performed using three-dimensional structure of *Torpedo californica* AChE (TcAChE) enzyme to disclose binding interaction and orientation of this molecule into the active site gorge of the receptor.

## 1. Introduction

Alzheimer's disease (AD) is associated with loss of cholinergic neurons in basal forebrain, which results in loss or failure of memory which slowly worsens and eventually incapacitates the patients [1, 2]. According to the World Alzheimer report, AD is one among the most significant social, health, and economic crises of the 21st century [3]. Although the exact factors initiating AD are unclear, genetic and environmental factors have been implicated [4]. In general, pharmacological therapies have twin objectives: (i) to prevent the loss of the neurons and (ii) to restore the cholinergic functions of AD patients. Cholinesterase inhibitors have been used clinically for symptomatic treatment of AD [5]. Acetylcholinesterase (AChE) enzyme is involved in the breakdown of acetylcholine in the brain and inhibition of this enzyme may increase the efficacy of treatment and broaden the indications [6]. Effects of cholinesterase inhibitors are mainly due to enhancement of cholinergic transmission at cholinergic autonomic synapses and at the neuromuscular junction [7].

AChE inhibitors are one of the most actively investigated classes of compounds in the search for an effective treatment of AD. Although there are many ongoing research activities in the search of drugs for treating AD, only few drugs like galantamine, donepezil, and rivastigmine are now available [8], and these drugs do not show potential cure rates; additional treatments are still being developed. The treatment of AD still remains an area of significant unmet need, with drugs that only target the symptoms of the disease. Therefore, there is considerable need for disease-modifying therapies. To meet the need of disease-modifying drugs for AD, in recent years, new approaches have emerged in medicinal chemistry. In particular the concept has recently been proposed that due to the multifactorial and complex etiology of AD, the modulation of a single factor might not be sufficient to produce the desired efficacy. Researchers are now paying attention to the design of structures that could be able to simultaneously interact with different targets involved in the pathogenic process [9, 10].

Naphthyridine structural motif has been extensively synthesized and incorporated into biologically active molecules [11]. In particular, 1,6-naphthyridines exhibit a broad spectrum of biological activities such as being inhibitor of HIV-1 integrase [12, 13], HCMV [14, 15], FGF receptor-1 tyrosine kinase [16], and the enzyme acetylcholinesterase [17]. Many routes for the syntheses of 1,6-naphthyridines derivatives have previously been reported [18–21]. In continuation of our previous work towards the synthesis of novel hybrid heterocycles employing new synthetic methodologies and/or their potential as inhibitors of AChE [22–26], herein we report the synthesis and AChE inhibitory activities of nitrogen heterocyclic hybrids comprising naphthyridine structural motif.

## 2. Materials and Methods

### 2.1. Chemistry

*General Methods.* Melting points were measured in KRUSS melting point meter using open capillary tubes and are uncorrected. ^1^H and ^13^C NMR spectra were recorded on a Bruker 300 MHz instrument in DMSO using TMS as internal standard. Standard Bruker software was used throughout. Chemical shifts are given in parts per million (*δ*-scale) and the coupling constants are given in Hertz. IR spectra were recorded in a Perkin Elmer system 2000 FT IR instrument (KBr). Elemental analyses were performed on a Perkin Elmer 2400 Series II Elemental CHNS analyser. Mass spectra were recorded in Agilent technologies 7820A GC-MS system.

#### 2.1.1. General Procedure for the Synthesis of Naphthyridines **(4a–k)**

A mixture of 3,5-bis[(*E*)-arylmethylidene]tetrahydro-4(1*H*)-pyridinones (1 mmol) and 2-cyanoacetamide (1 mmol) in ethanol (200 *μ*L) in the presence of catalytic amount of sodium ethoxide (5 mg) were ground well in a semimicro boiling tube at ambient temperature for about 6–10 min. After completion of the reaction as evident from TLC, water (50 mL) was added to the reaction mixture and the product was filtered, washed with water, and dried* in vacuo*.

*(E)-8-Benzylidene-2-oxo-4-phenyl-1,2,5,6,7,8-hexahydro-1,6-naphthyridine-3-carbonitrile (* ***4a*** *).* Pale yellow solid; IR (KBr) *ν*_max⁡_ 3372, 2945, 2213, 1642, 1625 cm^−1^; ^1^H NMR (300 MHz, DMSO): *δ*_H_ 3.15 (d,* J* = 15.9 Hz, 1H, 5-CH_2_), 3.35 (d,* J* = 15.9 Hz, 1H, 5-CH_2_), 3.60 (d,* J* = 15.6 Hz, 1H, 7-CH_2_), 3.69 (d,* J* = 15.6 Hz, 1H, 7-CH_2_), 7.07–7.38 (m, 10H, Ar-H), 7.81 (s, 1H, Arylmethylidene-H), 8.15 (s, 1H, NH). ^13^C NMR (75 MHz, DMSO): *δ*_C_ 45.48, 45.76, 79.31, 101.70, 115.22, 116.94, 126.21, 127.81, 128.84, 129.50, 130.78, 131.10, 134.71, 135.54, 137.90, 146.85, 159.18, 162.83. EIMS:* m/z* 341 [M+1]. Anal. calcd for C_22_H_17_N_3_O: C, 77.86; H, 5.05; N, 12.38; found: C, 77.69; H, 5.22; N, 12.25%.

*(E)-8-(2-Methylbenzylidene)-2-oxo-4-(o-tolyl)-1,2,5,6,7,8-hexahydro-1,6-naphthyridine-3-carbonitrile (* ***4b*** *).* Yellow solid; IR (KBr) *ν*_max⁡_ 3380, 2948, 2210, 1640, 1627 cm^−1^; ^1^H NMR (300 MHz, DMSO): *δ*_H_ 2.13 (s, 3H, CH_3_), 2.30 (s, 3H, CH_3_), 3.14 (d,* J* = 15.9 Hz, 1H, 5-CH_2_), 3.33 (d,* J* = 15.9 Hz, 1H, 5-CH_2_), 3.62 (d,* J* = 15.6 Hz, 1H, 7-CH_2_), 3.68 (d,* J* = 15.6 Hz, 1H, 7-CH_2_), 7.04–7.35 (m, 8H, Ar-H), 7.80 (s, 1H, Arylmethylidene-H), 8.14 (s, 1H, NH). ^13^C NMR (75 MHz, DMSO): *δ*_C_ 19.75, 20.56, 45.43, 45.82, 79.76, 101.32, 115.23, 116.91, 126.21, 127.03, 127.79, 128.89, 129.33, 129.53, 129.92, 130.78, 131.19, 134.76, 135.42, 135.55, 137.91, 146.96, 159.08, 162.92. EIMS:* m/z* 369 [M+1]. Anal. calcd for C_24_H_21_N_3_O: C, 78.45; H, 5.76; N, 11.44; found: C, 78.32; H, 5.98; N, 11.29%.

*(E)-8-(2-Chlorobenzylidene)-4-(2-chlorophenyl)-2-oxo-1,2,5,6,7,8-hexahydro-1,6-naphthyridine-3-carbonitrile (* ***4c*** *).* Pale yellow solid; IR (KBr) *ν*_max⁡_ 3384, 2950, 2214, 1642, 1625 cm^−1^; ^1^H NMR (300 MHz, DMSO): *δ*_H_ 3.16 (d,* J* = 15.6 Hz, 1H, 5-CH_2_), 3.34 (d,* J* = 15.6 Hz, 1H, 5-CH_2_), 3.61 (d,* J* = 15.6 Hz, 1H, 7-CH_2_), 3.68 (d,* J* = 15.6 Hz, 1H, 7-CH_2_), 7.10–7.36 (m, 8H, Ar-H), 7.81 (s, 1H, Arylmethylidene-H), 8.12 (s, 1H, NH). ^13^C NMR (75 MHz, DMSO): *δ*_C_ 45.38, 45.95, 79.70, 101.64, 115.21, 116.90, 126.28, 127.01, 127.85, 128.90, 129.31, 129.54, 129.93, 130.69, 131.26, 134.73, 135.40, 135.52, 137.90, 146.91, 159.13, 162.90. EIMS:* m/z* 408 [M+1]. Anal. calcd for C_22_H_15_Cl_2_N_3_O: C, 64.72; H, 3.70; N, 10.29; found: C, 64.80; H, 3.87; N, 10.48%.

*(E)-8-(2-Bromobenzylidene)-4-(2-bromophenyl)-2-oxo-1,2,5,6,7,8-hexahydro-1,6-naphthyridine-3-carbonitrile (* ***4d*** *).* Pale yellow solid; IR (KBr) *ν*_max⁡_ 3381, 2950, 2210, 1644, 1626 cm^−1^; ^1^H NMR (300 MHz, DMSO): *δ*_H_ 3.18 (d,* J* = 15.9 Hz, 1H, 5-CH_2_), 3.34 (d,* J* = 15.9 Hz, 1H, 5-CH_2_), 3.62 (d,* J* = 15.9 Hz, 1H, 7-CH_2_), 3.69 (d,* J* = 15.6 Hz, 1H, 7-CH_2_), 7.16–7.39 (m, 8H, Ar-H), 7.82 (s, 1H, Arylmethylidene-H), 8.09 (s, 1H, NH). ^13^C NMR (75 MHz, DMSO): *δ*_C_ 44.93, 45.80, 79.78, 101.61, 115.20, 116.96, 126.25, 127.05, 127.81, 128.98, 129.35, 129.62, 129.92, 130.73, 131.24, 134.71, 135.43, 135.51, 137.93, 146.90, 159.17, 162.92. EIMS:* m/z* 498 [M+1]. Anal. calcd for C_22_H_15_Br_2_N_3_O: C, 53.15; H, 3.04; N, 8.45; found: C, 53.38; H, 3.16; N, 8.37%.

*(E)-8-(2-Methoxybenzylidene)-4-(2-methoxyphenyl)-2-oxo-1,2,5,6,7,8-hexahydro-1,6-naphthyridine-3-carbonitrile (* ***4e*** *).* Yellow solid; IR (KBr) *ν*_max⁡_ 3385, 2944, 2210, 1641, 1627 cm^−1^; ^1^H NMR (300 MHz, DMSO): *δ*_H_ 3.14 (d,* J* = 15.6 Hz, 1H, 5-CH_2_), 3.31 (d,* J* = 15.6 Hz, 1H, 5-CH_2_), 3.60 (d,* J* = 15.6 Hz, 1H, 7-CH_2_), 3.68 (d,* J* = 15.6 Hz, 1H, 7-CH_2_), 3.74 (s, 3H, OCH_3_), 3.82 (s, 3H, OCH_3_), 7.11–7.38 (m, 8H, Ar-H), 7.83 (s, 1H, Arylmethylidene-H), 8.10 (s, 1H, NH). ^13^C NMR (75 MHz, DMSO): *δ*_C_ 45.39, 45.93, 55.51, 55.70, 79.70, 101.67, 114.20, 114.81, 115.20, 116.94, 126.31, 127.81, 128.93, 129.56, 130.66, 131.29, 134.70, 135.42, 137.90, 146.95, 159.07, 159.14, 160.04, 162.95. EIMS:* m/z* 401 [M+1]. Anal. calcd for C_24_H_21_N_3_O_3_: C, 72.16; H, 5.30; N, 10.52; found: C, 72.30; H, 5.53; N, 10.39%.

*(E)-8-(2,4-Dichlorobenzylidene)-4-(2,4-dichlorophenyl)-2-oxo-1,2,5,6,7,8-hexahydro-1,6-naphthyridine-3-carbonitrile (* ***4f*** *).* Pale yellow solid; IR (KBr) *ν*_max⁡_ 3380, 2951, 2219, 1646, 1628 cm^−1^; ^1^H NMR (300 MHz, DMSO): *δ*_H_ 3.18 (d,* J* = 15.9 Hz, 1H, 5-CH_2_), 3.37 (d,* J* = 15.9 Hz, 1H, 5-CH_2_), 3.60 (d,* J* = 15.6 Hz, 1H, 7-CH_2_), 3.68 (d,* J* = 15.6 Hz, 1H, 7-CH_2_), 7.14–7.42 (m, 6H, Ar-H), 7.82 (s, 1H, Arylmethylidene-H), 8.09 (s, 1H, NH). ^13^C NMR (75 MHz, DMSO): *δ*_C_ 45.35, 45.97, 79.65, 101.62, 115.26, 116.81, 126.30, 127.12, 127.87, 128.94, 129.43, 129.60, 129.94, 130.74, 131.27, 134.72, 135.47, 135.53, 137.94, 146.97, 159.16, 162.95. EIMS:* m/z* 478 [M+1]. Anal. calcd for C_22_H_13_Cl_4_N_3_O: C, 55.38; H, 2.75; N, 8.81; found: C, 55.29; H, 2.92; N, 8.70%.

*(E)-8-(3-Nitrobenzylidene)-4-(3-nitrophenyl)-2-oxo-1,2,5,6,7,8-hexahydro-1,6-naphthyridine-3-carbonitrile (* ***4g*** *).* Yellow solid; IR (KBr) *ν*_max⁡_ 3389, 2942, 2210, 1645, 1626 cm^−1^; ^1^H NMR (300 MHz, DMSO): *δ*_H_ 3.21 (d,* J* = 15.9 Hz, 1H, 5-CH_2_), 3.39 (d,* J* = 15.9 Hz, 1H, 5-CH_2_), 3.61 (d,* J* = 15.6 Hz, 1H, 7-CH_2_), 3.70 (d,* J* = 15.6 Hz, 1H, 7-CH_2_), 7.12–7.38 (m, 8H, Ar-H), 7.80 (s, 1H, Arylmethylidene-H), 8.11 (s, 1H, NH). ^13^C NMR (75 MHz, DMSO): *δ*_C_ 45.32, 45.95, 79.66, 101.60, 115.24, 116.79, 126.31, 127.15, 127.86, 128.93, 129.41, 129.64, 129.96, 130.75, 131.30, 134.71, 135.49, 135.61, 137.92, 146.95, 159.14, 162.93. EIMS:* m/z* 431 [M+1]. Anal. calcd for C_22_H_15_N_5_O_5_: C, 61.54; H, 3.52; N, 16.31; found: C, 61.27; H, 3.75; N, 16.18%.

*(E)-8-(4-Methylbenzylidene)-2-oxo-4-(p-tolyl)-1,2,5,6,7,8-hexahydro-1,6-naphthyridine-3-carbonitrile (* ***4h*** *).* Pale yellow solid; IR (KBr) *ν*_max⁡_ 3387, 2940, 2212, 1647, 1625 cm^−1^; ^1^H NMR (300 MHz, DMSO): *δ*_H_ 2.25 (s, 3H, CH_3_), 2.29 (s, 3H, CH_3_), 3.20 (d,* J* = 15.9 Hz, 1H, 5-CH_2_), 3.41 (d,* J* = 15.9 Hz, 1H, 5-CH_2_), 3.63 (d,* J* = 15.6 Hz, 1H, 7-CH_2_), 3.72 (d,* J* = 15.6 Hz, 1H, 7-CH_2_), 7.12–7.40 (m, 8H, Ar-H), 7.83 (s, 1H, Arylmethylidene-H), 8.12 (s, 1H, NH). ^13^C NMR (75 MHz, DMSO): *δ*_C_ 21.4, 21.8, 45.35, 45.97, 79.68, 101.64, 115.25, 116.80, 126.32, 127.18, 127.82, 128.91, 129.45, 129.96, 130.69, 131.35, 134.73, 135.47, 135.64, 137.90, 139.21, 146.94, 159.17, 162.90. EIMS:* m/z* 369 [M+1]. Anal. calcd for C_24_H_21_N_3_O: C, 78.45; H, 5.76; N, 11.44; found: C, 78.66; H, 5.87; N, 11.35%.

*(E)-8-(4-Chlorobenzylidene)-4-(4-chlorophenyl)-2-oxo-1,2,5,6,7,8-hexahydro-1,6-naphthyridine-3-carbonitrile (* ***4i*** *).* Yellow solid; IR (KBr) *ν*_max⁡_ 3385, 2942, 2210, 1645, 1627 cm^−1^; ^1^H NMR (300 MHz, DMSO): *δ*_H_ 3.17 (d,* J* = 15.9 Hz, 1H, 5-CH_2_), 3.40 (d,* J* = 15.9 Hz, 1H, 5-CH_2_), 3.62 (d,* J* = 15.6 Hz, 1H, 7-CH_2_), 3.71 (d,* J* = 15.6 Hz, 1H, 7-CH_2_), 7.16–7.48 (m, 8H, Ar-H), 7.87 (s, 1H, Arylmethylidene-H), 8.15 (s, 1H, NH). ^13^C NMR (75 MHz, DMSO): *δ*_C_ 45.39, 45.94, 79.70, 101.65, 115.26, 116.81, 126.34, 127.21, 127.85, 128.94, 129.48, 129.91, 130.76, 131.39, 134.71, 135.51, 135.60, 137.90, 146.92, 159.25, 162.97. EIMS:* m/z* 409 [M+1]. Anal. calcd for C_22_H_15_Cl_2_N_3_O: C, 64.72; H, 3.70; N, 10.29; found: C, 64.95; H, 3.84; N, 10.21%.

*(E)-8-(4-Fluorobenzylidene)-4-(4-fluorophenyl)-2-oxo-1,2,5,6,7,8-hexahydro-1,6-naphthyridine-3-carbonitrile (* ***4j*** *).* Pale yellow solid; IR (KBr) *ν*_max⁡_ 3387, 2940, 2213, 1645, 1624 cm^−1^; ^1^H NMR (300 MHz, DMSO): *δ*_H_ 3.15 (d,* J* = 15.9 Hz, 1H, 5-CH_2_), 3.37 (d,* J* = 15.9 Hz, 1H, 5-CH_2_), 3.63 (d,* J* = 15.6 Hz, 1H, 7-CH_2_), 3.70 (d,* J* = 15.9 Hz, 1H, 7-CH_2_), 7.13–7.45 (m, 8H, Ar-H), 7.84 (s, 1H, Arylmethylidene-H), 8.11 (s, 1H, NH). ^13^C NMR (75 MHz, DMSO): *δ*_C_ 45.37, 45.93, 79.72, 101.64, 114.36, 114.58, 115.23, 116.85, 126.37, 127.84, 128.92, 129.45, 130.72, 131.35, 134.78, 135.43, 137.94, 146.96, 159.25, 160.23, 161.18, 162.95. EIMS:* m/z* 377 [M+1]. Anal. calcd for C_22_H_15_F_2_N_3_O: C, 70.39; H, 4.03; N, 11.19; found: C, 70.65; H, 4.27; N, 11.10%.

*(E)-4-(Naphthalen-1-yl)-8-(naphthalen-1-ylmethylene)-2-oxo-1,2,5,6,7,8-hexahydro-1,6-naphthyridine-3-carbonitrile (* ***4k*** *).* Pale yellow solid; IR (KBr) *ν*_max⁡_ 3384, 2946, 2214, 1645, 1629 cm^−1^; ^1^H NMR (300 MHz, DMSO): *δ*_H_ 3.17 (d,* J* = 15.9 Hz, 1H, 5-CH_2_), 3.41 (d,* J* = 15.9 Hz, 1H, 5-CH_2_), 3.61 (d,* J* = 15.6 Hz, 1H, 7-CH_2_), 3.70 (d,* J* = 15.6 Hz, 1H, 7-CH_2_), 7.02–7.65 (m, 14H, Ar-H), 7.91 (s, 1H, Arylmethylidene-H), 8.17 (s, 1H, NH). ^13^C NMR (75 MHz, DMSO): *δ*_C_ 45.31, 45.84, 79.68, 101.65, 115.28, 116.80, 123.46, 124.03, 125.12, 125.57, 126.30, 126.42, 126.80, 127.20, 127.85, 128.22, 129.12, 129.48, 129.94, 130.79, 131.43, 132.10, 132.67, 134.73, 135.54, 135.68, 137.92, 146.90, 159.28, 162.94. EIMS:* m/z* 441 [M+1]. Anal. calcd for C_30_H_21_N_3_O: C, 81.98; H, 4.82; N, 9.56; found: C, 81.80; H, 4.95; N, 9.48%.

### 2.2. *In Vitro* Cholinesterase Enzymes Inhibitory Assay

Cholinesterase inhibitory activity of the synthesized compounds was evaluated using the Ellman's microplate assay [27]. For acetylcholinesterase (AChE) inhibitory assay, 140 *μ*L of 0.1 M sodium phosphate buffer (pH 8) was first added to a 96-well microplate followed by 20 *μ*L of test samples and 20 *μ*L of 0.09 units/mL acetylcholinesterase enzyme from* Electrophoruselectricus* (Sigma). After 15 minutes of incubation at 25°C, 10 *μ*L of 10 mM 5,5′-dithiobis-2-nitrobenzoic acid (DTNB) was added into each well followed by 10 *μ*L of acetylthiocholine iodide (14 mM). At 30 minutes after the initiation of enzymatic reaction, absorbance of the colored end-product was measured using BioTek Power Wave X 340 Microplate Spectrophotometer at 412 nm.

Galantamine was used as positive control. Test samples and galantamine were prepared in DMSO at an initial concentration of 1 mg/mL (1000 ppm). The concentration of DMSO in final reaction mixture was 1%. At this concentration, DMSO has no inhibitory effect on acetylcholinesterase enzyme.

The initial screening was carried out at 10 *μ*g/mL of test samples in 1% DMSO and each test was conducted in triplicate. Absorbencies of the test samples were corrected by subtracting the absorbance of their respective blank. Percentage enzyme inhibition is calculated using the following formula:

(1) Percentage  of  inhibition =Absorbance  of  sample−Absorbance  of  controlAbsorbance  of  control×100.

Subsequently, the determination of IC_50_ was carried out using a set of five concentrations.

### 2.3. Molecular Modeling

Using Glide (version 5.7, Schrödinger, LLC, New York, NY, 2011), most active compound was docked onto the active site of* Tc*AChE derived from three-dimensional structure of the enzyme complex with anti-Alzheimer's drug, galantamine (PDB ID: 4EVE).

Water molecules and hetero groups were deleted from enzyme beyond the radius of 5 Å of reference ligand (galantamine), resulting protein structure refined and minimized by Protein Preparation Wizard using OPLS-2005 force field. Receptor Grid Generation program was used to prepare* Tc*AChE grid and the ligand was optimized by LigPrep program by using OPLS-2005 force field to generate lowest energy state. Docking stimulations were carried out on bioactive compound, handed in 5 poses per ligand, in which the best pose with highest score was displayed for each ligand.

## 3. Results and Discussion

### 3.1. Chemistry

**Scheme 1:**
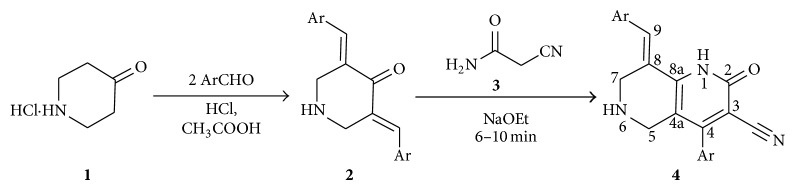
Synthesis of naphthyridines (**4a–k**).

In the present investigation, the reaction of a series of bisarylmethylidene piperidones with 2-cyanoacetamide in the presence of sodium ethoxide with few drops of ethanol under simple mixing at ambient temperature for 6–10 min afforded functionalized 1,6-naphthyridines in good yields (65–78%; Scheme 1). The prerequisite bisarylmethylidene piperidones were synthesized following the literature reported method [28]. In a typical reaction, an equimolar mixture of 3,5-bis[(*E*)-2-methylphenylmethylidene]tetrahydro-4(1*H*)-pyridinones (**2b**) and 2-cyanoacetamide (**3**) in catalytic amount of sodium ethoxide were ground well in a semimicro boiling tube with few drops of ethanol at ambient temperature for about 7 min and after completion of the reaction water was added to the mixture and the product was filtered and dried* in vacuo*. In this case, the 2-pyridone was obtained as a sole reaction product and does not require column chromatography for purification. Easy availability of the reagents, short reaction time, and simple reaction condition rendered this method more attractive from the viewpoint of green chemistry.

The structure of 1,6-naphthyridines was elucidated using IR, NMR, and CHN analysis. In the ^1^H NMR spectrum of** 4b**, the two doublets at 3.14 and 3.33 ppm with* J* = 15.9 Hz are due to 5-CH_2_ protons while the other doublets at 3.62 and 3.68 ppm with* J* = 15.6 Hz are due to 7-CH_2_ protons of the piperidine ring. The singlets at 8.14 and 7.80 ppm can be attributed to the NH of 2-pyridone ring and arylmethylidene proton, respectively. The two –CH_3_ protons of aromatic ring appear as singlets at 2.13 and 2.30 ppm while the multiplets around 7.04–7.35 ppm are due to aromatic protons. In the ^13^C NMR spectrum, the chemical shifts at 19.75 and 20.56 ppm were due to the two –CH_3_ carbons whilst the two methylene carbons of the piperidine ring resonated at 45.43 and 45.82 ppm. The aromatic carbons resonated at 101.32–162.92 ppm. The molecular ion peak at 369 [M+1] confirms the presence of compound** 4b**. The elemental analysis result of** 4b** was within ±0.4% of the theoretical values. The structure of other 2-pyridones was also assigned by similar considerations. Presumably, the 1,6-naphthyridine (**4**) is formed via a cascade heterocyclization mechanism involving an initial Michael addition followed by cyclization and air oxidation as reported by Jain et al. [29].

### 3.2. *In Vitro* Evaluation

All newly synthesized 1,6-naphthyridines were evaluated* in vitro* for their inhibitory potential against AChE enzyme from electric eel using colorimetric Ellman's method (Table 1). All the 1,6-naphthyridines (**4a–k**) displayed good to moderate inhibitory activities with IC_50_ values ranging from 2.12 to 24.72 *μ*M, irrespective of the position of the substituent on the aryl ring. Among the 1,6-naphthyridines, compounds** 4i** with* p*-chloro,** 4k** with 1-naphthyl,** 4h **with* p*-methyl, and** 4b** with* o*-methyl substituent in the aromatic ring displayed good activities (<10 *μ*M) with IC_50_ 3.86 *μ*M, 6.86 *μ*M, 7.16 *μ*M, and 7.20 *μ*M, respectively. Compounds** 4a**,** 4c**,** 4d**,** 4g,** and** 4j** displayed moderate activities (IC_50_ = 11.18–18.16 *μ*M) while compound** 4f** displayed the lowest activity (IC_50_ = 24.72 *μ*M). Compound** 4e** with* o-*methoxy phenyl rings displayed the highest activity with IC_50_ value of 2.12 *μ*M, comparable to the standard drug galantamine (IC_50_ = 2.09 *μ*M). It is also observed that the AChE inhibitory activities were directly correlated to the size of substituents in the phenyl ring. For instance, derivative bearing bulky moieties, such as* m*-nitro,* o*,*p*-dichloro, and* p*-bromo, displayed lower inhibition than the derivatives carrying smaller functions irrespective of their position in the phenyl ring.

The active site of AChE enzyme is located inside a 20 Å long, narrow gorge which is dominantly composed of amino acids possessing aromatic side chains such as tryptophan and tyrosine. Therefore, the derivatives bearing a relatively small and/or electron donating moieties in phenyl rings, such as methyl and methoxy, displayed better inhibitory activities than derivatives carrying bulky and/or electron withdrawing groups, plausibly due to the better insertion into the active site channel and also more efficient binding interaction with aforementioned aromatic residues. However with limited substituent on the aryl ring, it is difficult to ascertain the exact structure activity relationship based on their activities observed.

### 3.3. Docking Studies

**Figure 1:**
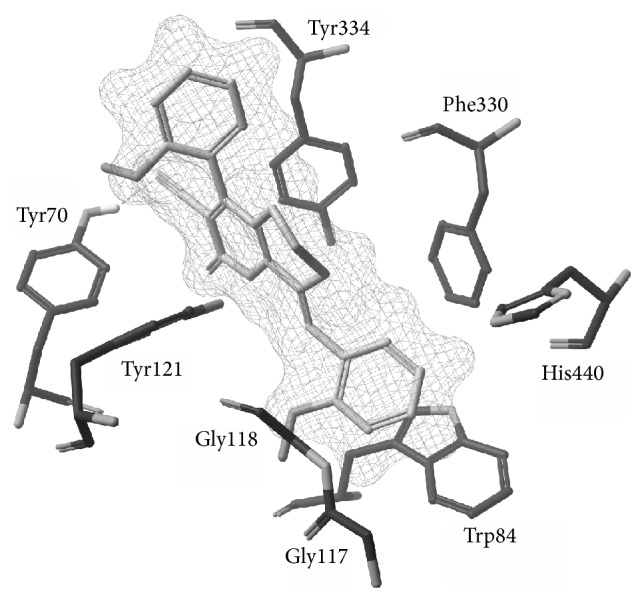
Binding interaction of** 4e** with active site of AChE receptor. (Hydrogen atoms are not shown for clarity.)

The most active AChE inhibitor,** 4e**, was docked into the active site of AChE enzyme derived from crystal structure of* Torpedo californica* AChE (*Tc*AChE). The docking analysis revealed that this compound is properly inserted into the active site of AChE enzyme with free binding energy of 8.71 kcal/mol and strongly bound to the residues comprising aromatic side chains such as Tyr70 (H-bonding 1.16 Å), Tyr121 (hydrophobic), Tyr334 (hydrophobic) at peripheral anionic site as well as Phe330 (hydrophobic), and Trp84 (*π*,*π*-stacking) at choline binding site of the enzyme (Figure 1).** 4e** also exhibited mild polar interaction with Gly116 and Gly117 at oxyanion hole of the AChE enzyme. The crystal structure of the* Tc*AChE in complex with available AD drugs such as galantamine and huperzine A showed similar interactions with residues composing peripheral anionic site along with stacking against Trp84 at bottom of the gorge. It seems that the presence of methoxy group in** 4e** has notable influence on proper positioning of this compound in AChE active site. This orientation effectively avoids insertion and hydrolysis of substrate inside the AChE active site channel and completely coincides with the activity observed for this compound.

## 4. Conclusion

A series of novel 1,6-naphthyridines were synthesized in good yields and evaluated for their inhibitory potentials against AChE enzyme, using colorimetric Ellman's method. Among them, compound** 4e** displayed the highest AChE inhibition with remarkable IC_50_ value of 2.12 *μ*M, comparable to standard drug, galantamine. Molecular modeling analysis for this compound manifested its orientation inside the active site cavity and its effective binding interactions to the residues lining the active site channel, which coincided with its* in vitro* activity.

## Figures and Tables

**Table 1:** Physical data and AChE inhibitory activity of naphthyridines (**4a–k**).

Entry	Product	Reaction time (min)	Yield (%)	mp °C	AChE inhibition (IC_50_ ± SD) mol/L
1	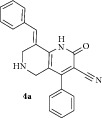	8	75	211-212	11.18 ± 0.02

2	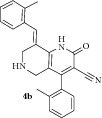	7	72	229-230	7.20 ± 0.02

3	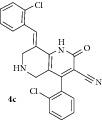	7	76	220-221	15.21 ± 0.1

4	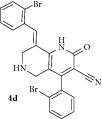	6	70	208-209	18.16 ± 0.02

5	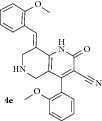	9	67	215-216	2.12 ± 0.02

6	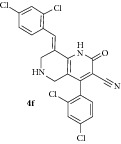	6	72	225-226	24.72 ± 0.01

7	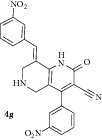	6	70	206-207	16.86 ± 0.02

8	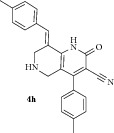	7	73	235-236	7.16 ± 0.02

9	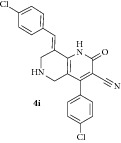	6	78	221-222	3.86 ± 0.02

10	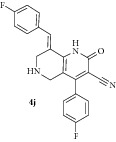	7	74	218-219	14.16 ± 0.02

11	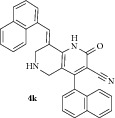	10	65	204-205	6.86 ± 0.02

12	Galantamine.HBr	—	—	—	2.09 ± 0.02
